# The role of tendon and ligament vasculature in ageing and injury

**DOI:** 10.1007/s10522-026-10491-4

**Published:** 2026-08-20

**Authors:** Nodoka Iwasaki, Elizabeth J. T. Finding, Caroline P. D. Wheeler-Jones, Chavaunne T. Thorpe

**Affiliations:** Comparative Biomedical Sciences, Royal Veterinary College, Royal College Street, London, NW1 0TU UK

**Keywords:** Ageing, Tendon, Ligament, Interfaces, Vasculature

## Abstract

Ageing is a fundamental biological process marked by declining physiological function and the accumulation of stress-induced damage. It contributes to chronic disease and drives widespread alterations across organ systems, including the vasculature. Ageing of the macro- and microvasculature impairs nutrient delivery, promotes tissue degeneration, and reduces repair capacity in many tissues, including those of the musculoskeletal system. Tendons and ligaments become increasingly injury-prone with age, yet the relationship between vascular ageing and tendon and ligament in health and disease remains insufficiently understood. This review critically examines the effects of vascular ageing on tendons and ligaments, including their interfaces with muscle and bone. Emerging evidence indicates that age-related vascular decline contributes to structural and functional impairments in these tissues, accelerating musculoskeletal deterioration. We also discuss therapeutic approaches targeting vascular dysfunction, including growth factor modulation through use of platelet-rich plasma, gene-based strategies, and stem cell therapies, which show potential for mitigating age-related deficits. By synthesizing existing knowledge, this review emphasizes the need for further research into vascular ageing and its implications for tendon and ligament health. A deeper understanding of these processes could inform the development of innovative therapies aimed at preserving vascular function and improving quality of life in the ageing population.

## Introduction

Ageing is a universal biological process that leads to a gradual and irreversible decline in physical function across organisms, driven by the accumulation of damage in response to various stressors (Guo et al. 2022). It is widely regarded as a major cause of many chronic diseases such as Alzheimer’s disease, cardiovascular disease, osteoporosis, and osteoarthritis, though its underlying mechanisms remain unclear (Fang et al. 2022). Understanding the mechanisms of ageing is crucial for identifying therapeutic targets for ameliorating age-related diseases and for developing new treatments.

Ageing induces many changes across all organ systems with significant alterations in the vasculature. Vascular ageing is characterised by several highly prevalent changes including arterial stiffening and calcification, which contribute to the decline in functional capacity in the elderly (El Assar et al. 2022). These age-related alterations affect both the macro- and microvasculature (Maier et al. 2023) and occur in the musculoskeletal system as well as other tissues (Roberts et al. 2016), leading to decreased quality of life in the elderly population. Musculoskeletal tissues affected by ageing include tendon and ligament, with increased injury risk and impaired reparative capacity evident with ageing (McCarthy and Hannafin 2014). However, the specific contribution of vascular ageing to age-related dysfunction in tendons and ligaments remains poorly defined.

This review discusses the alteration to tendon and ligament vasculature with ageing, including at their interfaces with muscle and bone, the effect of these changes and therapeutic approaches mitigating this alterations. Better understanding these processes is critical for developing therapeutic strategies to mitigate age-related functional decline and improve tissue function.

## Tendon vascular ageing

### Tendon structure and its vasculature

**Fig. 1:**
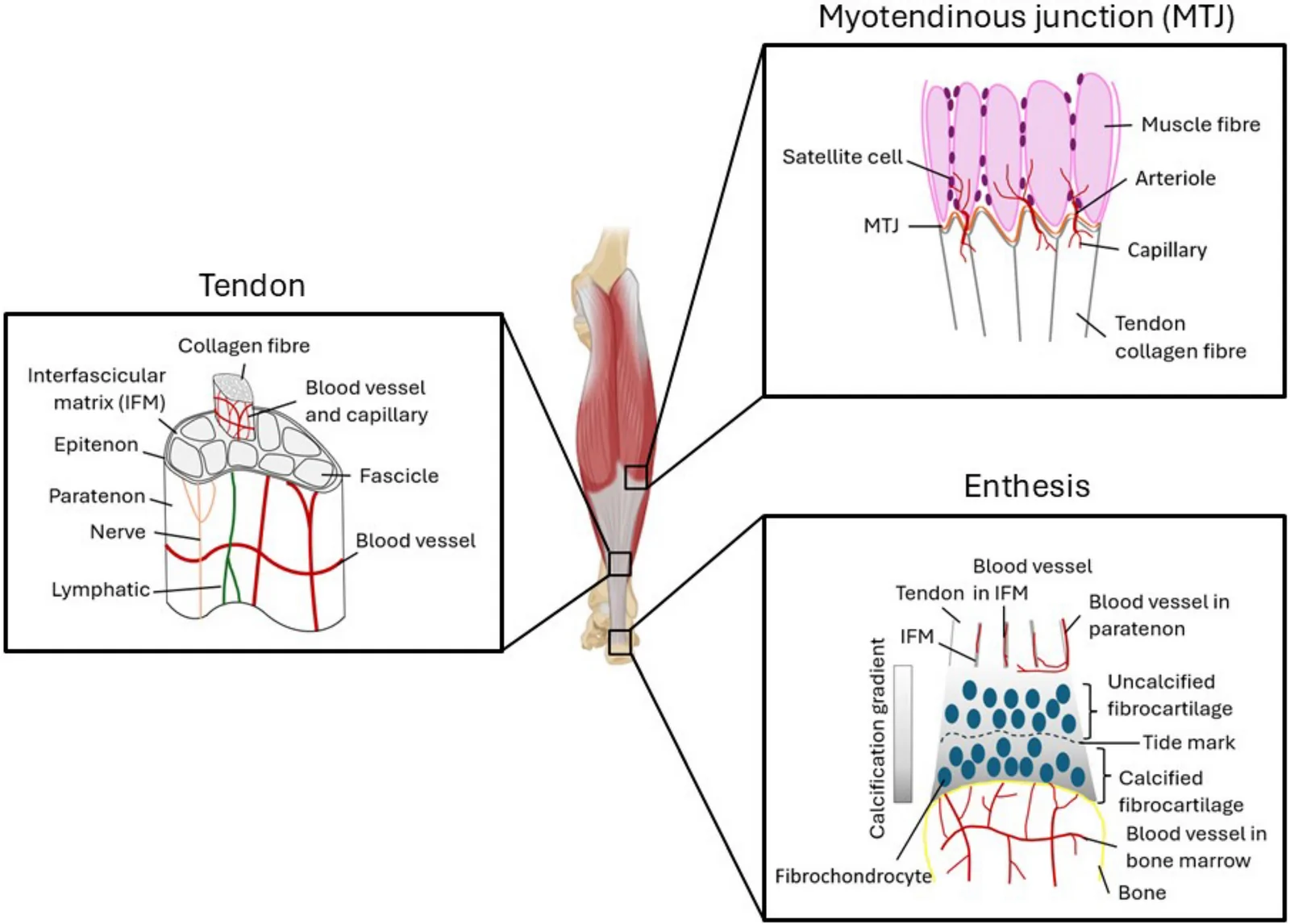
Schematic illustrations of the vasculature in tendon and interfaces

Tendon is a highly organised and dense connective tissue that connects muscles to bones and transmits muscle-generated force to the skeleton (Im and Kim 2020). Tendons play important roles in joint movement and in the maintenance of body posture (Bianchi et al. 2021). Their hierarchical structure is composed of collagen molecules, fibrils, fibres and fascicles, bound together by the interfascicular matrix (IFM, also referred to as endotenon) (Fig. 1). Surrounding each tendon is a layer of connective tissue called the paratenon, which carries blood vessels, lymphatics and nerves (Elliott 1965; Kannus 2000). Compared with muscles and bones, tendons are less vascularised, receiving blood from three sources: the muscle–tendon junction (MTJ), the osteotendinous junction (enthesis), and the paratenon (Ahmed et al. 1998) (Fig. 1). For many years, tendons were considered relatively avascular (Schmidt-Rohlfing et al. 1992; Ahmed et al. 1998). However, recent studies have shown that the IFM is rich in blood vessels, as evidenced by enrichment of vascular cell proteins such as CD146, CD31, VWF and desmin (Marr et al. 2023). Consistent with these findings, large numbers of endothelial cells and mural cells (vascular smooth muscle cells and pericytes) have been identified in this region using single-cell RNA sequencing and immunohistochemistry (Zamboulis et al. 2024). These IFM-localised blood vessels likely provide nutrients to cells within the surrounding fascicles.

### Vascular ageing in healthy tendon

Ageing is a common risk factor for tendon injury (McCarthy and Hannafin 2014; Korcari et al. 2023). Age-related alterations in tendon structure and function are well established, characterised by alterations in tendon mechanical properties, disorganisation of the extracellular matrix (ECM), and increases in collagen cross-linking (Svensson et al. 2016). However, the tendon vasculature has mainly been studied in the context of pathology, with few studies investigating the effects of vascular ageing in healthy tendons. The majority of these studies report that ageing has a detrimental effect on the tendon vasculature, with significantly reduced blood flow and microvascular density in human supraspinatus and gluteus medius tendons evident with ageing (Brewer 1979; Rudzki et al. 2008; Márquez-Arabia et al. 2017). It has also been shown that, while blood flow is reduced at rest in aged individuals, exercise-induced peritendinous blood flow in the Achilles region most prone to overuse injury did not change with age, suggesting that factors other than reduced blood flow during exercise contribute to the increased incidence of these injuries in older individuals (Langberg et al. 2001). Further, reduced ECM remodelling as well as decreased vascular density with ageing have been demonstrated in the rat Achilles tendon (Marqueti et al. 2018). Elastin, which localises to the IFM and is also essential for maintaining appropriate blood flow and pressure in arterial walls, decreased in the equine superficial digital flexor tendon (SDFT) with age (Godinho et al. 2017). Supporting these findings, recent work has shown a significant reduction in volume, diameter and density of larger blood vessels in the aged equine SDFT, measured using contrast-enhanced micro-computed tomography, but an increase in capillary density (Iwasaki et al. 2026). This suggests that while there is an overall decrease in vascularity in aged tendons, this is accompanied by neoangiogenesis; the mechanisms driving these processes, however, are yet to be determined (Iwasaki et al. 2026). Single cell RNA sequencing of young and old equine SDFTs also revealed that IFM-localised microvascular cells were affected by ageing, with dysregulation of genes associated with senescence and inflammation (Zamboulis et al. 2024). By contrast, an early study reported no change in vascular density with ageing in the equine SDFT, although this analysis was performed in 2D histological sections which may have missed changes apparent in 3D (Gillis et al. 1997). Similarly, no significant changes in rabbit flexor tendon blood flow were detected with ageing using the Xenon-133 washout technique, though comparisons were between young and mature, not mature and aged, animals (Landi et al. 1983).

While there are some inconsistent findings regarding the effects of ageing on the tendon microvasculature, most studies provide evidence that ageing has a detrimental effect on the tendon vasculature. Further investigation is required to improve understanding of how age-related alterations in the tendon vasculature contribute to loss of function and increased risk of injury with increasing age. Advanced high-resolution 3D imaging modalities, such as micro-computed tomography, are valuable tools for characterising structural changes in the tendon vasculature associated with ageing. In vitro vascularised tendon models are important for elucidating the cellular and molecular mechanisms underlying age-related changes in the tendon vasculature, but such models are yet to be fully developed and validated.

### Vascular ageing in injured/degenerated tendon

Tendon degeneration, or tendinopathy, is a common disorder, especially in the ageing population (McCarthy and Hannafin 2014). This can cause tendon thickening, loss of mechanical properties and pain, and may lead to rupture (Soslowsky et al. 2000; Andres and Murrell 2008; Gehwolf et al. 2025). Tendon regeneration is relatively poor, and risk of injury and degeneration increases with age (Teunis et al. 2014), thought to be due, at least in part, to the lower vascularity compared to muscle and bone (Docheva et al. 2015). Hypervascularisation is commonly observed in tendinopathy, with significantly increased neoangiogenesis in human Achilles tendinopathy biopsies compared to non-symptomatic areas of the same tendon (Aström and Rausing 1995; Merkel et al. 2021). There is also disorganised small-vessel ingrowth and endothelial cell hyperplasia in abnormal human patellar tendons (Yu et al. 1995), and large blood vessels are present in the paratenon in pathological human Achilles tendons, which may impair the gliding function of the paratenon (Aström and Rausing 1995). Nearly all patients with tendinopathy exhibit increased neovascularisation and tendon blood flow, as assessed by Doppler ultrasound (Merkel et al. 2021), and expression of the pro-angiogenic growth factor vascular endothelial growth factor (VEGF) is significantly increased in ruptured Achilles tendons (Pufe et al. 2001). However, VEGF was reported to be inversely correlated with tendon mechanical properties in a rat patellar injury model, suggesting that excessive VEGF-driven neovascularisation in healing tendons may negatively impact tendon health and mechanical properties (Sahin et al. 2012).

Traditionally, increased neovascularisaion was considered a classic pathological feature of tendinopathy (Alfredson and Cook 2007), which is induced by neurogenic inflammation to facilitate healing of micro-ruptures (van Sterkenburg and van Dijk 2011; Merkel et al. 2021). This concept is consistent with the longstanding view that inflammation drives angiogenesis as part of the tissue healing process following injury (Shi et al. 2023). However, this is not a consistent finding, with other studies showing no correlation between neovascularisation and pain or symptoms of tendinopathy (Tol et al. 2012; De Jonge et al. 2014).

Despite these associations, it is still unclear whether alterations to the tendon vasculature contribute to its degeneration and rupture, or whether degeneration results in disruption to the vasculature. It has been postulated that areas of reduced vascularity observed in several human tendons predispose these regions to degeneration and rupture (Schmidt-Rohlfing et al. 1992; Stein et al. 2000), but there is no direct evidence to support hypovascularity as a cause of tendon degeneration and rupture (Rathbun and Macnab 1970; Frey et al. 1990; Fenwick et al. 2002).

Ageing adds an additional layer of complexity. While neovascularisation is a key feature of injured and healing tendons, few studies have directly investigated the effect of vascular ageing on tendon injury and healing. Angiogenesis following injury is reduced in aged rat tendons, with decreased blood flow and vessel density compared to young animals, which may contribute to poor tendon healing potential with ageing (Leahy et al. 2022; Riggin et al. 2022).

Reduced vascularity or blood flow are not the only factors linked to impaired healing. Other factors affecting healing include deposition of disorganised collagen, scar tissue development, inadequate rehabilitation or immobilisation, overloading, and surgical interventions (Graham and Nwankwo 2025). For instance, several studies have demonstrated that overloading can cause tissue damage, which results in vascular cell recruitment and hypervascularity at the injury site due to a failed healing response (Arnoczky et al. 2007; Moschini et al. 2026).

In summary, tendinopathy is consistently associated with pathological hypervascularisation, yet evidence suggests that aged tendons mount a diminished angiogenic response following injury. These apparently divergent findings highlight the complexity of tendon vascular biology. Tendon regeneration and vascularisation are complex and establishing the precise alterations in the angiogenic response in ageing tendons remains a key area for future research. Comparative studies assessing angiogenic responses in healthy young versus aged tendons, as well as in tendinopathy and injury models, will be particularly valuable for determining whether ageing, tendinopathy, or injury are the primary drivers of angiogenic changes in tendon.

### Therapeutic approaches targeting and affecting the tendon vasculature

Various therapeutic approaches have been investigated to improve tendon healing, with growing evidence suggesting that some exert their effects, at least in part, by modulating angiogenesis and tendon vascularity (Table 1). One of the most common treatments for tendon injuries and degeneration is physiotherapy, but few studies exist regarding its effect on the tendon vasculature. While moderate treadmill running performed prior to injury promoted healing of surgically induced patellar tendon lesions in aged rats, it also reduced vascularisation (Zhang et al. 2016). However, vascularisation was only assessed macroscopically on the tendon surface, so any changes in neovascularisation within the tendon tissue itself could not be assessed. Another study in a rat model found that the age-related decrease in vascular density within the Achilles tendon was ameliorated by resistance training, suggesting that this exercise modality may help prevent tendon vascular age-associated degeneration (Marqueti et al. 2018). These findings suggest that the impact of physiotherapy on tendon vascularisation remains unclear, and that various forms of exercise may influence the tendon vasculature in different ways.

**Table 1:** Details of species and outcomes of therapeutic treatments in tendons

Treatment	Species	Age	Outcome	References
Physiotherapy	Rat	20 months old	Moderate treadmill running performed prior to injury promoted healing of injured patellar tendon and reduced vascularisation	Zhang et al. (2016)
	Rat	3 months old (young) and 20 months old (aged)	Resistance training ameliorated age-related decrease in vascular density in the Achilles tendon	Marqueti et al. (2018)
Growth factor therapy	Rat	Information not available	VEGF treatment improved tensile strength in Achilles tendon healing	Zhang et al. (2003)
	Rat	2 months old	VEGF-111 treatment improves the early phases of the healing process	Kaux et al. (2014)
	Rat	4 months old (young) and 16 months old (aged)	VEGF treatment increased vascular response to tendon injury and improved mechanical outcome in this aged population	Riggin et al. (2022)
Antibody treatment	Rat	4 months old	VEGF treatment showed minor changes but anti-VEGF antibody treatment increased healing and tendon vascularity	Riggin et al. (2021)
	Rat	2 months old	Antibody treatment to block VEGF signalling pathway reduced angiogenesis and increased healing after injury	Tempfer et al. (2018)
Photobiomodulation therapy	Rat	12 weeks old (young) and 18 months old (aged)	Photobiomodulation therapy upregulated VEGF expression in aged rats with induced tendinopathy	Marques et al. (2016)
Gene therapy	Chicken	Information not available	VEGF or bFGF gene therapy increased tendon strength in injured tendon	Tang et al. (2016)
	Horse	6–15 years old	VEGF and FGF gene therapy showed significant ultrasonographic and clinical improvements	Kovac et al. (2018)
PRP treatment	Human	39.5 ± 6.9 years old (young) and 61.5 ± 5.3 years old (aged)	PRP treatment in aged patients with Achilles tendinopathy was less effective than in young patients	Salini et al. (2015)
	Human	< 50 years (young) and > 50 years (aged)	Platelet lysate concentrations from aged donors needed to be higher to increase tenocyte proliferation and migration compared to younger donors	Berger et al. (2019)
BMC treatment	Human	49–71 years old	The use of BMC containing MSC as an adjunct therapy significantly improved rotator cuff healing and tendon integrity	Hernigou et al. (2014)
	Human	38.3 ± 9.6 years old	The use of BMAC in conjunction with open Achilles tendon repair showed no re-rupture and early mobilisation	Stein et al. (2015)
SVF treatment	Human	47.8 ± 5.1 years old	SVF treatment resulted in a significant reduction of pain and an increase in tendon thickness in Achilles tendinopathy patients	Albano et al. (2017)
	Human	18–55 years old	SVF treatment demonstrated significantly better healing in Achilles tendons compared to PRP treatment at early timepoints	Usuelli et al. (2018)
	Rabbit	Information not available	SVF treatment improved tendon healing, associated with a decrease in neovascularisation	Behfar et al. (2011)
Stem cell therapy	Rabbit	Information not available	Bone marrow derived MSC therapy showed a higher cell density and vascularization in tendons	Vaquette et al. (2010)
	Rat	Information not available	Human MSC injections enhanced tendon healing through improved vascularisation and ECM organisation in Achilles tendon injury model	Machova Urdzikova et al. (2014)
EV treatment	Rat	Information not available	Bioactive glass pre-treated MSC-EVs showed improved therapeutic capacity with enhanced angiogenesis during tendon regeneration	Xu et al. (2023)
	Mouse	10 weeks old	Adipose stem cell derived EVs reduced vascularity and promoted healing in chronic rotator cuff tendinopathy model	Wang et al. (2021)

There is also an interest in developing therapeutic approaches for age-related tendon degeneration by restoring or enhancing physiological vascular functions in tendons, with many focusing on VEGF, which has an important role in chronic inflammation and wound healing by inducing endothelial cell proliferation and new blood vessel formation (Angelo and Kurzrock 2007). Treatment with VEGF following injury caused increased blood flow and oxygenation, and improved mechanical behaviour in the healing adult and aged rat Achilles tendon (Zhang et al. 2003; Kaux et al. 2014; Riggin et al. 2022). However, some studies have shown contradictory results. For example, it was reported that VEGF treatment had minor effects on healing and vascular changes in rat Achilles tendon at the doses tested, but use of a VEGF antibody that prevents VEGF from interacting with its receptors, increased vascularity and healing later in the repair process (Riggin et al. 2021). Another study using an antibody that blocks VEGF signalling showed reduced vascularity in rat Achilles tendon and increased healing after injury (Tempfer et al. 2018).

Photobiomodulation therapy, which promotes tissue repair using a low power laser, upregulated VEGF expression in aged rats with induced tendinopathy, and increased collagen type I and III deposition (Marques et al. 2016). VEGF or basic fibroblast growth factor (bFGF) gene therapy, where VEGF or bFGF genes were transferred using single-stranded AAV2 vectors, also resulted in significant improvement in collagen type I production and tendon strength in injured chicken tendons (Tang et al. 2016), and significant clinical improvements in 8 out of 10 horses with a naturally occurring tendon injury (Kovac et al. 2018).

Another therapy that targets the vasculature is intralesional administration of platelet-rich plasma (PRP); concentrated autologous plasma containing various growth factors, including VEGF and FGF (Fréchette et al. 2005; Pneumaticos et al. 2011). PRP is commonly used to treat tendinopathy in humans (Van Schaik and Lee 2021), improving pain and function in human patients (Rha et al. 2013; Chaudhary et al. 2016; Niazi et al. 2020; Prodromos et al. 2021). However, its efficacy in promoting tendon healing appears to vary by anatomical site, with more favourable outcomes reported for patellar tendons than for Achilles tendons (Filardo et al. 2018), and some studies have shown no differences between PRP-treated groups and control (placebo) groups (Dai et al. 2023). In addition, the effects of PRP appear to decrease with ageing, with reduced efficacy in Achilles tendinopathy in patients over the age of 60 compared to young and middle-aged patients (Salini et al. 2015). Higher platelet lysate concentrations from aged donors are also needed to increase tenocyte proliferation and migration compared to younger donors (Berger et al. 2019). This may reflect alterations in PRP composition with age, since the proteomic profile of PRP from older people differs from that in young humans (Jiang et al. 2024). In addition, PRP isolated from older horses contains reduced concentrations of platelet-derived growth factor isoform BB (Giraldo et al. 2013).

Bone marrow concentrate (BMC), containing mesenchymal stem cells (MSCs) and growth factors such as VEGF, has also been used in human patients with tendinopathy and has shown promising results, improving tendon integrity and preventing re-tears (Hernigou et al. 2014; Stein et al. 2015). More recently, adipose-derived stromal vascular fraction (SVF), containing pre-adipocytes, vascular endothelial cells, smooth muscle cells, leukocytes, erythrocytes and pericytes, has been used to treat human tendon injuries, resulting in a significant reduction of pain and an increase in tendon thickness (Albano et al. 2017). SVF-treated human tendons also demonstrated significantly better healing compared to PRP treated tendons at early timepoints (Usuelli et al. 2018). While these therapeutics contain vascular cells and associated factors and are therefore likely to affect the tendon vasculature, no direct association between vascular cell function/vascularisation and tendon healing has been reported in human studies. However, a small number of animal studies have shown that stem cell therapy increases tendon vascularisation, as assessed histologically, in tendons with surgically induced injuries (Vaquette et al. 2010; Machova Urdzikova et al. 2014). By contrast, SVF-treated rabbit Achilles tendons demonstrated improved healing, associated with a decrease in neovascularisation (Behfar et al. 2011).

Extracellular vesicles (EVs) have been introduced to promote tendon healing more recently. Bioactive glass pre-treated EVs derived from MSCs (MSC-EVs) showed improved therapeutic capacity with enhanced angiogenesis evident during rat tendon regeneration compared to non-treated MSC-EVs (Xu et al. 2023). Adipose stem cell derived EVs reduced vascularity in chronic rotator cuff tendinopathy in mice and promoted healing (Wang et al. 2021).

Several treatments for tendinopathy and tendon injuries are associated with effects on the vasculature, but more work is required to enhance knowledge of how these treatments impact healing, and how this is influenced by ageing. High-throughput approaches such as multi-omic analyses combined with in vitro vascularised tendon models would provide a powerful approach to comprehensively characterise age- and treatment-related vascular responses and the mechanisms driving these responses; however, such models require development.

Given the role of the vasculature as a regulator of tissue repair, increasing attention has focused on the perivascular niche as a potential source of regenerative cells. Tendon stem/progenitor cells (TSPCs), which have the potential to differentiate into tenocytes, play an important role in tendon repair and several studies have shown improved tendon healing with TSPC therapy (Lu et al. 2023). Evidence suggests that TSPCs are derived from the perivascular niche within tendons, and perivascular niche-derived cells have been shown to contribute to regeneration in a variety of tissues (Tempfer et al. 2009). These findings suggest that the perivascular niche may represent a promising therapeutic target for promoting tendon repair and treating tendon degeneration.

## Ligament vascular ageing

### Ligament structure and its vasculature

Ligament is a connective tissue that connects bone to bone and limits joint motion (Hauser et al. 2013; Leong et al. 2020), with a hierarchical structure similar to tendon (Fig. 1) (Lake et al. 2020). Ligament can be classified either as intra-articular, such as the anterior cruciate ligament (ACL), or extra-articular, such as the medial collateral ligament (MCL) (McCarthy and Hannafin 2014). Both intra-articular and extra-articular ligaments are covered by a connective tissue layer called the epiligament, which contains blood vessels comprised of endothelial cells and mural cells, and additional cells including ligament fibroblasts and adipocytes (Chowdhury et al. 1991). The ligaments associated with the knee joint, including the ACL and MCL, are the most well studied due to their relatively high risk of injury (Rao et al. 2022). The ACL is vascularised by branches of the lateral and medial inferior geniculate artery and has an avascular region in the central portion (Petersen and Tillmann 1999). A study using canine ACL showed that these vessels originate from the synovium surrounding the ligament and penetrate the ACL near its femoral and tibial insertions to form an intrinsic vascular network (Kobayashi et al. 2006). Unlike the ACL, the MCL is relatively well-vascularised with blood supply from branches of the superior and inferior geniculate arteries (Juneja and Hubbard 2025). High magnification histology has demonstrated numerous capillaries within the MCL, whereas the ACL contains comparatively few capillaries (Wallace and Amiel 1991).

### Vascular ageing in healthy and injured/degenerated ligament

Compared with tendon vasculature, ligament vasculature has received substantially less research attention, and comprehensive studies investigating the effects of ageing on ligament vascularity are currently lacking. As ligaments age, they undergo compositional changes such as decreased collagen organisation, alterations in ECM composition and cell activity (e.g. stem cell properties), as well as increased calcification, which collectively affect their mechanotransduction, healing capacity, and biomechanical function (McCarthy and Hannafin 2014; Boros and Freemont 2017; Hudson et al. 2021). However, few studies to date have investigated the effects of ageing on the ligament vasculature. Tohno and co-workers reported age-related iron loss in human ACL, which was attributed to reduced blood supply, despite a lack of direct evidence to support this (Tohno et al. 1999). There is also limited evidence that expression and regulation of vascular-related genes are altered with ageing, with increased expression of vascular ageing-related microRNAs (miR-29a and miR-34a) in old mice compared to younger animals (Kharaz et al. 2022). Another study demonstrated a decrease in growth factor receptor expression with age, including FGF receptor 2 and VEGF receptor 2, in porcine ACL fibroblasts, although this study compared young with adolescent animals (Vavken et al. 2010).

Increasing age is also a risk factor for ligament injury, particularly for the ACL, in which injuries in those over 40 are increasing (Mall et al. 2014). There is also some evidence that injury and healing capacity are associated with an altered vascularisation and altered vascular cell functions in aged ligaments. Inflammation is one factor associated with ligament degeneration, and it is characterised by neovascularisation in the ligament sheath, which has shown to be positively correlated with donor age in the ACL (Hasegawa et al. 2012). Injured ACLs are not only more vascularised (Takeuchi et al. 2022), but also contain more vascular-derived stem cells compared to the non-injured mid-substance region, suggesting that vascular stem cells contribute to ligament healing (Matsumoto et al. 2012). However, stem cells in ligament lose their expansion and differentiation potential with age, which likely limits their reparative capacity (Uefuji et al. 2014). There are also differences in blood flow, vascular volume, and inflammation-induced angiogenesis between ligament types with injury; the MCL has a greater capacity to increase blood flow and angiogenesis after injury than the ACL, which likely contributes to the greater healing capacity observed in the MCL (Woo et al. 2000; Bray et al. 2003).

### Therapeutic approaches affecting and targeting the ligament vasculature

For MCL injuries, non-surgical treatments such as physiotherapy are the most common treatment (Miyamoto et al. 2009), a strategy likely facilitated by the ligament’s relatively high vascularity, which supports greater regenerative capacity and intrinsic healing compared with the ACL (Bray et al. 2002; Lucidi et al. 2024). However, no studies have investigated the effect of physiotherapy on ligament vasculature. Due to its limited healing capacity, surgical intervention is 4 times more common than non-surgical treatments in ACL injuries in the UK (Beard et al. 2024). However, reconstructed ligaments may not be fully restored, which affects patients’ quality of life (Kvist et al. 2014; Grevnerts et al. 2021).

There are also several non-surgical techniques used to treat ligament injuries that likely affect the vasculature (Table 2). Growth factors and PRP are commonly used to treat ligament, as well as tendon, injuries. In human clinical studies, grafts with PRP gel have been reported to shorten ACL healing time by ~ 48% compared to controls (Radice et al. 2010). However, a systematic review has reported that while PRP may reduce pain and improve knee function in the short to medium term following ACL reconstruction, these benefits do not appear to be sustained over the long term (Zhu et al. 2022). Similarly, another review found no consistent evidence that PRP enhances graft healing after ACL reconstruction, suggesting that its overall effect on ACL healing remains limited (Cao and Wan 2022).

**Table 2:** Details of species and outcomes of therapeutic treatments in ligaments

Treatment	Species	Age	Outcome	References
PRP treatment	Human	18–35 years old	Grafts with PRP shortened ACL healing time by 48% compared to controls	Radice et al. (2010)
Growth factor treatment	Rat	8 weeks old	VEGF treatment induced capillary formation in the injured rat MCL and showed significantly greater functional healing	Nishimori et al. (2012)
	Dog	Information not available	FGF treatment enhanced neovascularisation and improved healing in the ACL	Kobayashi et al. (1997)
Stem cell therapy	Rat	Information not available	MSC treatment improved MCL healing with enhanced blood vessel formation	Saether et al. (2016)
	Rabbit	3 months old	MSCs in collagen scaffold increased blood vessel formation at injured ACL	Figueroa et al. (2014)
	Rat	Information not available	Different doses of MSCs uniquely affected the cellular response and blood vessel formation in MCL	Saether et al. (2014)
	Rat	8–12 weeks old	CD34+ cell treatment increased endothelial cell marker expression and capillary density in injured MCL	Tei et al. (2008)
	Rat	Information not available	CD34+ cell treatment induced neovascularisation at injury site in MCL	Jiang et al. (2015)

Administration of exogenous VEGF induced capillary formation in the injured rat MCL, and ligaments with higher capillary density showed significantly greater functional healing (Nishimori et al. 2012). Similarly, treatment with bFGF enhanced neovascularisation and improved healing in the canine ACL with surgically induced defects (Kobayashi et al. 1997), suggesting that angiogenesis plays an important role in ligament healing.

Growth factors have also been used to improve the function of allografts and autografts for ligament repair (Ju et al. 2006; Yoshikawa et al. 2006; Chen et al. 2012; Xie et al. 2013). PRP, which contains several growth factors, induced revascularisation of canine ACL autografts (Xie et al. 2013), while VEGF promoted angiogenesis and revascularisation in rabbit ACL autografts (Ju et al. 2006) and in rabbit tendon allografts used to repair the ACL (Chen et al. 2012).

Stem cell therapy has also been used to treat degenerated ligaments as well as tendons, and MSCs are frequently incorporated alongside grafts or scaffolds. Several studies have reported that MSC therapy induces vascularisation in healing ligaments, both with and without scaffolds. For example, MSCs injected into injured rat MCL co-localised with endothelial cells and pericytes, and MSC-treated ligaments showed enhanced blood vessel formation, suggesting that MSCs may induce angiogenesis (Saether et al. 2016). It was also demonstrated that MSCs within a collagen type-I scaffold enhance blood vessel formation in injured rabbit ACL compared to the suture-treated or scaffold-treated ligaments without MSCs (Figueroa et al. 2014). In addition, it has been reported that MSC treatment can alter the number of endothelial cells and blood vessel formation in injured rat MCL, which correlates with improved mechanical properties and early healing, in a dose-dependent manner (Saether et al. 2014). Umbilical cord blood-derived CD34+ cells is another cell type that has been used to treat degenerated ligaments. They are reported to differentiate into endothelial cells in vivo (Tei et al. 2008), and induce neovascularisation at the injury site in rat MCLs (Jiang et al. 2015).

While it is evident that several treatments for ligament injury affect the vasculature, little is known about how this is affected by ageing, and this remains an important area for future research. As proposed earlier for tendon, omics-based approaches combined with in vitro vascularised ligament models incorporating induced ageing, or in vivo animal models could be employed to further investigate the mechanisms underlying age-related changes in ligament regeneration.

## Tendon and ligament interfaces

### Structure and vascularisation of interfaces

Despite the high rates of injury to tendon and ligament interfaces, relatively little is known about the vascularity of tendon and ligament interface tissues, and the effects of ageing on the vasculature of healthy or injured interfaces remain unexplored. Tendons have two interface regions: between tendon and muscle, the myotendinous junction (MTJ), and between tendon and bone, the enthesis, while ligaments have an enthesis at either end (Fig. 1). The MTJ is an anatomically specialised interface between muscle and tendon, which transmits the force generated by the muscle to the tendon (Tidball 1983), whereas an enthesis is specialised tissue consisting of several transitional zones to enable smooth transduction of mechanical forces between tendon/ligament and bone (De Lorenzis et al. 2023). The MTJ has a complex structure with finger-like processes composed of overlapping muscle and tendon fibres (Bayrak and Yilgor Huri 2018; Tong et al. 2024). It forms a gradient from the myofiber, through a transitional zone and tip at the MTJ, to the tendon MTJ, and finally the tendon proper (Mackey 2024). The MTJ has a capillary-arteriole-capillary vascular system, where capillaries merge into small arterioles, the arterioles penetrate through the MTJ and then branch into capillaries on the other side (Kvist et al. 1995) (Fig. 1). The enthesis also has a complex structure, consisting of four transitional zones: dense fibrous connective tissue (tendon or ligament), uncalcified fibrocartilage, calcified fibrocartilage, and bone (Benjamin et al. 2002; Mata Arnaiz and de Miguel Mendieta 2014; Schett et al. 2017; Tsechelidis et al. 2023). The blood supply to the enthesis is mainly from vessels in the bone marrow and tendon/ligament (Fig. 1), which can penetrate the enthesis (Benjamin and McGonagle 2001; Morel et al. 2005). However, there is a complete lack of vascularisation at the bone attachment site and within the uncalcified and calcified fibrocartilage (Watad et al. 2018; Tsechelidis et al. 2023). Overall, blood vessels are found mainly at the interfaces of both tendons and ligaments (Ahmed et al. 1998; Wang et al. 2006).

Regeneration of the enthesis after injury occurs by forming fibrovascular scar tissue, which lacks gradient mineral distribution and organised collagen fibre orientation, resulting in a weak tissue that is more prone to injury (Font Tellado et al. 2015; Lei et al. 2021). Increased vascularity was found in the inflamed enthesis (McGonagle et al. 2002), and vascular channel localisation was positively correlated with inflammatory and osteoclast activated sites, suggesting that vascular channels may be a key marker for identifying inflammation and degenerative arthritis within the enthesis (Binks et al. 2015).

### Therapeutic approaches affecting and targeting vasculature of the interfaces

Injuries at the enthesis are common, especially in the rotator cuff (Long et al. 2022), and surgical intervention is a standard treatment for tendon and ligament enthesis repair; however, it causes fibrovascular scar tissue formation between the tendon/ligament and the bone, which lowers the mechanical properties of the enthesis and increases the risk of re-tearing (Derwin et al. 2018). Tears at the MTJ are mainly caused by sporting activities (Noonan and Garrett 1999; Aicale et al. 2018), and they can also occur secondarily to tendon or muscle injuries; however, relatively few studies have reported the treatment of MTJ tears (Millett et al. 2017; Recker et al. 2021; Giordano et al. 2022). Surgical intervention is the only established treatment for a complete tear at the MTJ, but satisfactory long-term outcomes following surgery are compromised by the formation of adhesions and a decline in mechanical properties, which collectively heighten the risk of re-rupture (Yan et al. 2018).

By contrast, stem cell therapy is emerging as a potential therapeutic for enthesis injuries (Table 3), and various types of stem cells are reported to improve enthesis healing, including BMC/bone marrow-derived MSCs (Ouyang et al. 2004; Hernigou et al. 2014; Liu et al. 2018), human umbilical cord blood-derived MSCs (Jang et al. 2015), and ACL-derived CD34+ cells (Mifune et al. 2012; Mifune et al. 2013). ACL-derived CD34+ cells differentiate into endothelial cells and osteoblasts in vivo, and CD34+ cell injections induce greater angiogenesis and healing in rat enthesis than control injections (Mifune et al. 2012). In addition, CD34+ cell sheets enhanced neovascularisation and capillary density in rat tendon enthesis to a greater extent than cell injections (Mifune et al. 2013). It has also been reported that ACL-derived CD34+ cells from younger patients (10–19 years) facilitated better healing in rat enthesis than cells from older patients (30–39 years) (Nakano et al. 2015).

**Table 3:** Details of species and outcomes of therapeutic treatments in the enthesis

Treatment	Species	Age	Outcome	References
Stem cell therapy	Rat	10 weeks old	ACL-derived CD34+ cell therapy enhanced angiogenesis and healing at the enthesis	Mifune et al. (2012)
	Rat	Information not available	CD34+ cell sheets enhanced neovascularisation and capillary density	Mifune et al. (2013)
	Rat	10 weeks old	ACL derived CD34+ cells from younger human patients (10–19 years) showed better healing capacity	Nakano et al. (2015)
Growth factor treatment	Rat	Information not available	TGF-β3 increased the vascularity and healing of enthesis	Manning et al. (2011)
	Rabbit	12 weeks old	A combination of PRP and MSCs promoted healing	Teng et al. (2016)
EV treatment	Rat	4 weeks old	MSC-EVs improved angiogenesis and healing after rotator cuff reconstruction	Huang et al. (2020)
BPC-157 treatment	Rat	Information not available	BPC-157 treatment improved vascularity and healing	Krivic et al. (2006)

Growth factors, as well as PRP, can also enhance healing potential in the enthesis (Manning et al. 2011; Zhang et al. 2019; Chen et al. 2022); transforming growth factor beta 3 (TGF-β3) increased the vascularity and healing of rat tendon enthesis (Manning et al. 2011) and a combination of PRP and bone marrow-derived MSCs promoted healing in rabbit tendon enthesis (Teng et al. 2016). Similarly, EVs derived from MSCs (MSC-EVs) promoted angiogenesis and healing after rotator cuff reconstruction in rat enthesis (Huang et al. 2020).

For MTJ repair and regeneration, tissue engineering approaches have also been investigated (Table 4). This approach, however, has its challenges due to the complex structure of the MTJ, the different mechanical properties of the tissues involved, and the cellular differences between tendon and muscle (Arrigoni et al. 2019). For example, xenogeneic ECM was used in resected dog MTJ and formed vascularised and innervated muscle; however, the contractile force generated by the engineered MTJ was lower than the force generated by the native MTJ (Turner et al. 2010). Skeletal muscle-derived stem cell sheets have also been used to treat complete tears of the MTJ in mice, and the engrafted cells predominantly developed into connective tissues and neurovascular networks, forming bridges between ruptured tendon and muscle fibres (Hashimoto et al. 2016). Lineage differentiation into skeletal muscle fibres, Schwann cells, vascular smooth muscle cells, and endothelial cells was observed; however, tensile testing of the regenerated tissues was not conducted, leaving their mechanical properties undetermined (Hashimoto et al. 2016).

**Table 4:** Details of species and outcomes of therapeutic treatments in the MTJ

Treatment	Species	Age	Outcome	References
ECM-based scaffold	Dog	Information not available	ECM-based scaffold formed vascularised and innervated muscle at the MTJ	Turner et al. (2010)
Stem cell therapy	Mouse	8–12 weeks old	Skeletal muscle-derived stem cell sheets formed neovascular network and bridges between ruptured tendon and muscle fibres	Hashimoto et al. (2016)
BPC-157 treatment	Rat	12 weeks old	BPC-157 treatment increased vascularity with capillary penetration and improved healing	Japjec et al. (2021)

In other studies, Body Protection Compound-157 (BPC-157), a synthetic peptide known to heal and repair damaged muscle, tendon and ligament, was used in the defective rat MTJ and resulted in increased vascularity with capillary penetration at the MTJ, and improved MTJ healing (Japjec et al. 2021). BPC-157 has also been reported to improve vascularity and healing in the rat enthesis (Krivic et al. 2006). BCP-157 therapy is non-toxic, does not require a carrier, and seems to be a promising treatment for soft tissue healing. Further investigation is now required to determine the mechanisms driving the improved healing response (Japjec et al. 2021). However, these findings are derived from studies on animal models, and robust evidence from human clinical studies on BPC-157 efficacy is still lacking (Mayfield et al. 2026).

While there are a range of potential therapeutics for interface injuries that appear to influence the vasculature, the relationship between vascular changes and healing remains undefined, as does the effect of ageing on the vasculature in healthy and diseased interfaces.

## Conclusions

While ageing and injury-associated changes to the vasculature in tendons and ligaments remain understudied, there is accumulating evidence that both tendons and ligaments exhibit reduced vascularisation with increasing age, which likely contributes to structural degeneration, increased risk of injury and reduced healing capacity. However, age-related alterations in the vasculature of the MTJ and enthesis, and subsequent influence on reparative capacity remain undefined. This review highlights the critical need for comprehensive research to elucidate the complex interactions between vascular ageing and tendon/ligament health, particularly at their interfaces. While therapeutic strategies targeting vascular dysfunction, such as growth factor-based approaches including the use of PRP, gene therapies and stem cells show promise, identifying the mechanisms through which these therapeutics provide benefit is essential to refine treatments and translate them into effective clinical interventions. Indeed, enhanced understanding of the mechanisms underlying vascular ageing and regeneration will ultimately contribute to the development of targeted, multidisciplinary therapeutics for tendon and ligament health preservation in older adults.

## Data Availability

No datasets were generated or analysed during the current study.

## References

[CR1] Ahmed IM, Lagopoulos M, McConnell P, Soames RW, Sefton GK (1998) Blood supply of the achilles tendon. J Orthop Res 16(5):591–596 10.1002/jor.1100160511 9820283

[CR2] Aicale R, Tarantino D, Maffulli N (2018) Overuse injuries in sport: a comprehensive overview. J Orthop Surg Res 13(1):1–12. 10.1186/s13018-018-1017-5 PMC6282309 30518382

[CR3] Albano D et al (2017) Magnetic resonance and ultrasound in achilles tendinopathy: predictive role and response assessment to platelet-rich plasma and adipose-derived stromal vascular fraction injection. Eur J Radiol 95:130–135. 10.1016/j.ejrad.2017.08.006 28987658

[CR4] Alfredson H, Cook J (2007) A treatment algorithm for managing Achilles tendinopathy: new treatment options: figure 1. Br J Sports Med 41(4):211–216. 10.1136/bjsm.2007.035543 PMC2658946 17311806

[CR5] Andres BM, Murrell GAC (2008) Treatment of tendinopathy: what works, what does not, and what is on the horizon. Clin Orthop Relat Res 466(7):1539–1554. 10.1007/s11999-008-0260-1 PMC2505250 18446422

[CR6] Angelo LS, Kurzrock R (2007) Vascular endothelial growth factor and its relationship to inflammatory mediators. Clin Cancer Res 13(10):2825–2830 10.1158/1078-0432.CCR-06-2416 17504979

[CR7] Arnoczky SP, Lavagnino M, Egerbacher M (2007) The mechanobiological aetiopathogenesis of tendinopathy: is it the over-stimulation or the under-stimulation of tendon cells? Int J Exp Pathol. 10.1111/j.1365-2613.2007.00548.x PMC2517314 17696902

[CR8] Arrigoni C, Petta D, Bersini S, Mironov V, Candrian C, Moretti M (2019) Engineering complex muscle-tissue interfaces through microfabrication. Biofabrication 11(3):032004 10.1088/1758-5090/ab1e7c 31042682

[CR10] Aström M, Rausing A (1995) Chronic Achilles tendinopathy. A survey of surgical and histopathologic findings. Clin Orthop Relat Res 316:151–164 7634699

[CR11] Bayrak E, Yilgor Huri P (2018) Engineering musculoskeletal tissue interfaces. Front Mater 5(April):1–8. 10.3389/fmats.2018.00024

[CR12] Beard DJ et al (2024) Comparison of surgical or non-surgical management for non-acute anterior cruciate ligament injury: the ACL SNNAP RCT. Health Technol Assess. 10.3310/VDKB6009 PMC11228690 38940695

[CR13] Behfar M, Sarrafzadeh-Rezaei F, Hobbenaghi R, Delirezh N, Dalir-Naghadeh B (2011) Adipose-derived stromal vascular fraction improves tendon healing in rabbits. Chin J Traumatol = Zhonghua Chuang Shang Za Zhi. 14(6):329–335 22152135

[CR14] Benjamin M, McGonagle D (2001) The anatomical basis for disease localisation in seronegative spondyloarthropathy at entheses and related sites. J Anat 199(5):503–526 10.1046/j.1469-7580.2001.19950503.x PMC1468363 11760883

[CR15] Benjamin M, Kumai T, Milz S, Boszczyk BM, Boszczyk AA, Ralphs JR (2002) The skeletal attachment of tendons—tendon ‘entheses.’ Comp Biochem Physiol a: Mol Integr Physiol 133(4):931–945. 10.1016/S1095-6433(02)00138-1 12485684

[CR16] Berger DR, Centeno CJ, Steinmetz NJ (2019) Platelet lysates from aged donors promote human tenocyte proliferation and migration in a concentration-dependent manner. Bone Joint Res 8(1):32–40. 10.1302/2046-3758.81.BJR-2018-0164.R1 PMC6359887 30800297

[CR17] Bianchi E et al (2021) Innovative strategies in tendon tissue engineering. Pharmaceutics. 10.3390/pharmaceutics13010089 PMC7827834 33440840

[CR18] Binks DA et al (2015) Role of vascular channels as a novel mechanism for subchondral bone damage at cruciate ligament entheses in osteoarthritis and inflammatory arthritis. Ann Rheum Dis 74(1):196–203. 10.1136/annrheumdis-2013-203972 PMC4283693 24095939

[CR19] Boros K, Freemont T (2017) Physiology of ageing of the musculoskeletal system. Best Pract Res Clin Rheumatol 31(2):203–217. 10.1016/j.berh.2017.09.003 29224697

[CR20] Bray RC, Leonard CA, Salo PT (2002) Vascular physiology and long‐term healing of partial ligament tears. J Orthop Res 20(5):984–989. 10.1016/S0736-0266(02)00012-8 12382963

[CR21] Bray RC, Leonard CA, Salo PT (2003) Correlation of healing capacity with vascular response in the anterior cruciate and medial collateral ligaments of the rabbit. J Orthop Res 21(6):1118–1123. 10.1016/S0736-0266(03)00078-0 14554227

[CR22] Brewer BJ (1979) Aging of the rotator cuff. Am J Sports Med 7(2):102–110. 10.1177/036354657900700206 434288

[CR23] Cao Y, Wan Y (2022) Effectiveness of platelet‐rich plasma in anterior cruciate ligament reconstruction: a systematic review of randomized controlled trials. Orthop Surg 14(10):2406–2417 10.1111/os.13279 PMC9531067 36056588

[CR24] Chaudhary, D., Rohit, K. and Kalla, R. 2016. Medical science functional outcome and results of platelet rich plasma (Prp). In rotator cuff tendinopathy.

[CR25] Chen J, Yang L, Guo L, Duan X (2012) Sodium hyaluronate as a drug-release system for VEGF 165 improves graft revascularization in anterior cruciate ligament reconstruction in a rabbit model. Exp Ther Med 4(3):430–434. 10.3892/etm.2012.629 PMC3503698 23181113

[CR26] Chen R, Zhu H, Gu X, Xiang X (2022) Effects of platelet-rich plasma on tendon-bone healing after anterior cruciate ligament reconstruction. Orthop Surg 14(1):88–95. 10.1111/os.13175 PMC8755887 34870370

[CR27] Chowdhury R, Matyas JR, Frank CB (1991) The “epiligament” of the rabbit medial collateral ligament: a quantitative morphological study. Connect Tissue Res 27(1):33–50. 10.3109/03008209109006993 1773613

[CR28] Dai W, Yan W, Leng X, Wang J, Hu X, Cheng J, Ao Y (2023) Efficacy of platelet-rich plasma versus placebo in the treatment of tendinopathy: a meta-analysis of randomized controlled trials. Clin J Sport Med 33(1):69–77. 10.1097/JSM.0000000000000961 34342296

[CR29] de F. Marques AC et al (2016) Photobiomodulation therapy on collagen type I and III, vascular endothelial growth factor, and metalloproteinase in experimentally induced tendinopathy in aged rats. Lasers Med Sci 31(9):1915–1923 10.1007/s10103-016-2070-0 27624782

[CR30] Derwin KA, Galatz LM, Ratcliffe A, Thomopoulos S (2018) Enthesis repair. J Bone Joint Surg 100(16):e109. 10.2106/JBJS.18.00200 PMC6133216 30106830

[CR31] Docheva D, Müller SA, Majewski M, Evans CH (2015) Biologics for tendon repair. Adv Drug Deliv Rev 84:222–239. 10.1016/j.addr.2014.11.015 PMC4519231 25446135

[CR9] El Assar M, Álvarez-Bustos A, Sosa P, Angulo J, Rodríguez-Mañas L (2022) Effect of physical activity/exercise on oxidative stress and inflammation in muscle and vascular aging. Int J Mol Sci. 10.3390/ijms23158713 PMC9369066 35955849

[CR32] Elliott DH (1965) Structure and function of mammalian tendon. Biol Rev 40(3):392–421 10.1111/j.1469-185x.1965.tb00808.x 14340913

[CR33] Fang H, Deng Z, Liu J, Chen S, Deng Z, Li W (2022) The mechanism of bone remodeling after bone aging. Clin Interv Aging 17:405–415. 10.2147/CIA.S349604 PMC8994557 35411139

[CR34] Fenwick SA, Hazleman BL, Riley GP (2002) The vasculature and its role in the damaged and healing tendon. Arthritis Res 4(4):252–260 10.1186/ar416 PMC128932 12106496

[CR35] Figueroa D, Espinosa M, Calvo R, Scheu M, Vaisman A, Gallegos M, Conget P (2014) Anterior cruciate ligament regeneration using mesenchymal stem cells and collagen type I scaffold in a rabbit model. Knee Surg Sports Traumatol Arthrosc 22(5):1196–1202. 10.1007/s00167-013-2471-6 23474696

[CR36] Filardo G, Di Matteo B, Kon E, Merli G, Marcacci M (2018) Platelet-rich plasma in tendon-related disorders: results and indications. Knee Surg Sports Traumatol Arthrosc 26(7):1984–1999. 10.1007/s00167-016-4261-4 27665095

[CR37] Font Tellado S, Balmayor ER, Van Griensven M (2015) Strategies to engineer tendon/ligament-to-bone interface: biomaterials, cells and growth factors. Adv Drug Deliv Rev 94:126–140. 10.1016/j.addr.2015.03.004 25777059

[CR38] Fréchette J-P, Martineau I, Gagnon G (2005) Platelet-rich plasmas: growth factor content and roles in wound healing. J Dent Res 84(5):434–439. 10.1177/154405910508400507 15840779

[CR39] Frey C, Shereff M, Greenidge N (1990) Vascularity of the posterior tibial tendon. J Bone Joint Surg Am 72(6):884–888 2195033

[CR40] Gehwolf R, Tempfer H, Cesur NP, Wagner A, Traweger A, Lehner C (2025) Tendinopathy: the interplay between mechanical stress, inflammation, and vascularity. Adv Sci. 10.1002/advs.202506440 PMC12463082 40855996

[CR41] Gillis C, Pool RR, Meagher DM, Stover SM, Reiser K, Willits N (1997) Effect of maturation and aging on the histomorphometric and biochemical characteristics of equine superficial digital flexor tendon. Am J Vet Res 58(4):425–430. 10.2460/ajvr.1997.58.04.425 9099392

[CR42] Giordano J, Partan M, Iturriaga C, Granata J, Katsigiorgis G, Cohn R, Bitterman A (2022) The relationship between patient demographics, tear locations, and operative techniques on the surgical treatment of acute achilles tendon ruptures. Cureus. 10.7759/cureus.28300 PMC9506559 36168374

[CR43] Giraldo CE, López C, Álvarez ME, Samudio IJ, Prades M, Carmona JU (2013) Effects of the breed, sex and age on cellular content and growth factor release from equine pure-platelet rich plasma and pure-platelet rich gel. BMC Vet Res 9(1):29. 10.1186/1746-6148-9-29 PMC3577464 23402541

[CR44] Godinho MSC, Thorpe CT, Greenwald SE, Screen HRC (2017) Elastin is Localised to the interfascicular matrix of energy storing tendons and becomes increasingly disorganised with ageing. Sci Rep. 10.1038/s41598-017-09995-4 PMC5577209 28855560

[CR45] Graham R, Nwankwo CD (2025) An overview of tendon physiology: the impact of injury and disease on structure, function and healing. Orthop Trauma 39(5):302–307

[CR46] Grevnerts HT, Sonesson S, Gauffin H, Ardern CL, Stålman A, Kvist J (2021) Decision making for treatment after ACL injury from an orthopaedic surgeon and patient perspective: results from the NACOX study. Orthop J Sports Med. 10.1177/23259671211005090 PMC8053763 33948447

[CR47] Guo J, Huang X, Dou L, Yan M, Shen T, Tang W, Li J (2022) Aging and aging-related diseases: from molecular mechanisms to interventions and treatments. Signal Transduct Target Ther. 10.1038/s41392-022-01251-0 PMC9755275 36522308

[CR48] Hasegawa A et al (2012) Anterior cruciate ligament changes in the human knee joint in aging and osteoarthritis. Arthritis Rheum 64(3):696–704. 10.1002/art.33417 PMC3266452 22006159

[CR49] Hashimoto H, Tamaki T, Hirata M, Uchiyama Y, Sato M, Mochida J (2016) Reconstitution of the complete rupture in musculotendinous junction using skeletal muscle-derived multipotent stem cell sheet-pellets as a ‘bio-bond.’ PeerJ 4:e2231. 10.7717/peerj.2231 PMC4957990 27547541

[CR50] Hauser RA, Dolan EE, Phillips HJ, Newlin AC, Moore RE, Woldin BA (2013) Ligament injury and healing: a review of current clinical diagnostics and therapeutics. Open Rehabil J 6(1):1–20

[CR51] Hernigou P, Flouzat Lachaniette CH, Delambre J, Zilber S, Duffiet P, Chevallier N, Rouard H (2014) Biologic augmentation of rotator cuff repair with mesenchymal stem cells during arthroscopy improves healing and prevents further tears: a case-controlled study. Int Orthop 38(9):1811–1818. 10.1007/s00264-014-2391-1 24913770

[CR52] Huang Y, He B, Wang L, Yuan B, Shu H, Zhang F, Sun L (2020) Bone marrow mesenchymal stem cell-derived exosomes promote rotator cuff tendon-bone healing by promoting angiogenesis and regulating M1 macrophages in rats. Stem Cell Res Ther. 10.1186/s13287-020-02005-x PMC7687785 33239091

[CR53] Hudson DM, Archer M, Rai J, Weis M, Fernandes RJ, Eyre DR (2021) Age-related type I collagen modifications reveal tissue-defining differences between ligament and tendon. Matrix Biol plus 12:100070. 10.1016/j.mbplus.2021.100070 PMC8605237 34825162

[CR54] Im GI, Kim TK (2020) Stem cells for the regeneration of tendon and ligament: a perspective. Int J Stem Cells 13(3):335–341. 10.15283/IJSC20091 PMC7691853 33122471

[CR55] Iwasaki N, Llewellyn J, Brown J, Zamboulis DE, Finding EJT, Wheeler-Jones CPD, Thorpe CT (2026) Immunolabelling and micro-computed tomography revealed age-related alterations in 3d microvasculature of tendons. Aging Cell. 10.1111/acel.70293 PMC12740099 41250917

[CR56] Jang K-M, Lim HC, Jung WY, Moon SW, Wang JH (2015) Efficacy and safety of human umbilical cord blood–derived mesenchymal stem cells in anterior cruciate ligament reconstruction of a rabbit model: new strategy to enhance tendon graft healing. Arthroscopy 31(8):1530–1539. 10.1016/j.arthro.2015.02.023 25882182

[CR57] Japjec M et al (2021) Stable gastric pentadecapeptide BPC 157 as a therapy for the disable myotendinous junctions in rats. Biomedicines (Basel) 9(11):1547. 10.3390/biomedicines9111547 PMC8615275 34829776

[CR58] Jiang D, Yang S, Gao P, Zhang Y, Guo T, Lin H, Geng H (2015) Combined effect of ligament stem cells and umbilical-cord-blood-derived CD34+ cells on ligament healing. Cell Tissue Res 362(3):587–595. 10.1007/s00441-015-2250-4 26224540

[CR59] Jiang Z et al (2024) Platelet-rich plasma in young and elderly humans exhibits a different proteomic profile. J Proteome Res 23(5):1788–1800. 10.1021/acs.jproteome.4c00030 38619924

[CR60] De Jonge S et al (2014) Relationship between neovascularization and clinical severity in <scp>A</scp> chilles tendinopathy in 556 paired measurements. Scand J Med Sci Sports 24(5):773–778. 10.1111/sms.12072 23600756

[CR61] Ju Y-J, Tohyama H, Kondo E, Yoshikawa T, Muneta T, Shinomiya K, Yasuda K (2006) Effects of local administration of vascular endothelial growth factor on properties of the in situ frozen-thawed anterior cruciate ligament in rabbits. Am J Sports Med 34(1):84–91. 10.1177/0363546505278700 16210580

[CR62] Juneja, P. and Hubbard, J.B. 2025. Anatomy, bony pelvis and lower limb, knee medial collateral ligament. 29939557

[CR63] Kannus P (2000) Structure of the tendon connective tissue. Scand J Med Sci Sports 10(6):312–320. 10.1034/j.1600-0838.2000.010006312.x 11085557

[CR64] Kaux J-F et al (2014) Vascular Endothelial Growth Factor-111 (VEGF-111) and tendon healing: preliminary results in a rat model of tendon injury. Muscles Ligaments Tendons J 4(1):24–28 PMC4049645 24932443

[CR65] Kharaz YA, Goljanek-Whysall K, Nye G, Hurst JL, McArdle A, Comerford EJ (2022) Age-related changes in microRNAs expression in cruciate ligaments of wild-stock house mice. Physiol Rep. 10.14814/phy2.15426 PMC9393909 35993414

[CR66] Kobayashi D, Kurosaka M, Yoshiya S, Mizuno K (1997) Effect of basic fibroblast growth factor on the healing of defects in the canine anterior cruciate ligament. Knee Surg Sports Traumatol Arthrosc 5(3):189–194. 10.1007/s001670050049 9335032

[CR67] Kobayashi S et al (2006) Microvascular system of anterior cruciate ligament in dogs. J Orthop Res 24(7):1509–1520. 10.1002/jor.20183 16732615

[CR68] Korcari A, Przybelski SJ, Gingery A, Loiselle AE (2023) Impact of aging on tendon homeostasis, tendinopathy development, and impaired healing. Connect Tissue Res 64(1):1–13 10.1080/03008207.2022.2102004 PMC9851966 35903886

[CR69] Kovac M, Litvin YA, Aliev RO, Zakirova EY, Rutland CS, Kiyasov AP, Rizvanov AA (2018) Gene therapy using plasmid DNA encoding VEGF164 and FGF2 genes: a novel treatment of naturally occurring tendinitis and desmitis in horses. Front Pharmacol. 10.3389/fphar.2018.00978 PMC6127648 30233367

[CR70] Krivic A, Anic T, Seiwerth S, Huljev D, Sikiric P (2006) Achilles detachment in rat and stable gastric pentadecapeptide BPC 157: promoted tendon-to-bone healing and opposed corticosteroid aggravation. J Orthop Res 24(5):982–989. 10.1002/jor.20096 16583442

[CR71] Kvist M, Hurme T, Kannus P, Järvinen T, Maunu V-M, Jozsa L, Järvinen M (1995) Vascular density at the myotendinous junction of the rat gastrocnemius muscle after immobilization and remobilization. Am J Sports Med 23(3):359–364 10.1177/036354659502300320 7661268

[CR72] Kvist J, Kartus J, Karlsson J, Forssblad M (2014) Results from the Swedish National Anterior Cruciate Ligament Register. Arthroscopy 30(7):803–810. 10.1016/j.arthro.2014.02.036 24746404

[CR73] Lake SP, Liu Q, Xing M, Iannucci LE, Wang Z, Zhao C (2020) Tendon and ligament tissue engineering. Principles of tissue engineering. Elsevier, pp 989–1005. 10.1016/B978-0-12-818422-6.00056-3

[CR74] Landi A, Elves M, Piaggi W (1983) The blood flow of rabbits’ tendons: variation with age activity hypoxia. Acta Orthop Scand 54(6):832–835 10.3109/17453678308992917 6670506

[CR75] Langberg H, Olesen J, Skovgaard D, Kjær M (2001) Age related blood flow around the Achilles tendon during exercise in humans. Eur J Appl Physiol 84(3):246–248. 10.1007/s004210170013 11320644

[CR76] Leahy TP, Nuss CA, Evans MK, Fung AK, Shetye SS, Soslowsky LJ (2022) Achilles tendon ruptures in middle-aged rats heal poorly compared with those in young and old rats. Am J Sports Med 50(1):170–181. 10.1177/03635465211055476 PMC8819270 34851182

[CR77] Lei T, Zhang T, Ju W, Chen X, Heng BC, Shen W, Yin Z (2021) Biomimetic strategies for tendon/ligament-to-bone interface regeneration. Bioact Mater 6(8):2491–2510. 10.1016/j.bioactmat.2021.01.022 PMC7889437 33665493

[CR78] Leong NL, Kator JL, Clemens TL, James A, Enamoto-Iwamoto M, Jiang J (2020) Tendon and ligament healing and current approaches to tendon and ligament regeneration. J Orthop Res : off Pub Orthop Res Soc 38(1):7–12. 10.1002/jor.24475 PMC7307866 31529731

[CR79] Liu XN et al (2018) Enhanced tendon-to-bone healing of chronic rotator cuff tears by bone marrow aspirate concentrate in a rabbit model. Clin Orthop Surg 10(1):99. 10.4055/cios.2018.10.1.99 PMC5851862 29564054

[CR80] Long Z, Nakagawa K, Wang Z, Amadio PC, Zhao C, Gingery A (2022) Age‐related cellular and microstructural changes in the rotator cuff enthesis. J Orthop Res 40(8):1883–1895. 10.1002/jor.25211 PMC9107523 34783060

[CR81] De Lorenzis E, Natalello G, Simon D, Schett G, D’Agostino MA (2023) Concepts of entheseal pain. Arthritis Rheumatol 75(4):493–498. 10.1002/art.42299 35818681

[CR82] Lu J et al (2023) The functions and mechanisms of tendon stem/progenitor cells in tendon healing. Stem Cells Int. 10.1155/2023/1258024 PMC10509002 37731626

[CR83] Lucidi GA, Solaro L, Grassi A, Alhalalmeh MI, Ratti S, Manzoli L, Zaffagnini S (2024) Current trends in the medial side of the knee: not only medial collateral ligament (MCL). J Orthop Traumatol. 10.1186/s10195-024-00808-9 PMC11662134 39704918

[CR84] Machova Urdzikova L et al (2014) Human multipotent mesenchymal stem cells improve healing after collagenase tendon injury in the rat. Biomed Eng Online 13(1):42. 10.1186/1475-925X-13-42 PMC4001357 24712305

[CR85] Mackey AL (2024) The myotendinous junction—form and function. Cold Spring Harb Perspect Biol. 10.1101/cshperspect.a041500 PMC12401052 39134380

[CR86] Maier JA et al (2023) Aging and vascular disease: a multidisciplinary overview. J Clin Med 12(17):5512 10.3390/jcm12175512 PMC10488447 37685580

[CR87] Mall NA, Chalmers PN, Moric M, Tanaka MJ, Cole BJ, Bach BR, Paletta GA (2014) Incidence and trends of anterior cruciate ligament reconstruction in the United States. Am J Sports Med 42(10):2363–2370. 10.1177/0363546514542796 25086064

[CR88] Manning CN, Kim HM, Sakiyama‐Elbert S, Galatz LM, Havlioglu N, Thomopoulos S (2011) Sustained delivery of transforming growth factor beta three enhances tendon‐to‐bone healing in a rat model. J Orthop Res 29(7):1099–1105. 10.1002/jor.21301 21246611

[CR89] Marqueti RC et al (2018) Effects of aging and resistance training in rat tendon remodeling. FASEB J 32(1):353–368. 10.1096/fj.201700543r 28899880

[CR90] Márquez-Arabia WH et al (2017) Influence of aging on microvascular supply of the gluteus medius tendon: a cadaveric and histologic study. Arthroscopy 33(7):1354–1360. 10.1016/j.arthro.2017.01.036 28390662

[CR91] Marr N, Zamboulis DE, Werling D, Felder AA, Dudhia J, Pitsillides AA, Thorpe CT (2023) The tendon interfascicular basement membrane provides a vascular niche for CD146+ cell subpopulations. Front Cell Develop Biol. 10.3389/fcell.2022.1094124 PMC9869387 36699014

[CR92] Mata Arnaiz MC, de Miguel Mendieta E (2014) Usefulness of ultrasonography in the assessment of peripheral enthesis in spondyloarthritis. Reumatología Clínica (English Edition) 10(2):113–119. 10.1016/j.reumae.2013.11.008 24360900

[CR93] Matsumoto T et al (2012) Isolation and characterization of human anterior cruciate ligament-derived vascular stem cells. Stem Cells Dev 21(6):859–872. 10.1089/scd.2010.0528 PMC3871494 21732814

[CR94] Mayfield CK et al (2026) Injectable peptide therapy: a primer for orthopaedic and sports medicine physicians. Am J Sports Med 54(1):223–229. 10.1177/03635465251357593 41476424

[CR95] McCarthy MM, Hannafin JA (2014) The mature athlete. Sports Health 6(1):41–48. 10.1177/1941738113485691 PMC3874221 24427441

[CR96] McGonagle D, Marzo-Ortega H, O’Connor P, Gibbon W, Hawkey P, Henshaw K, Emery P (2002) Histological assessment of the early enthesitis lesion in spondyloarthropathy. Ann Rheum Dis 61(6):534–537. 10.1136/ard.61.6.534 PMC1754106 12006328

[CR97] Merkel MFR, Hellsten Y, Magnusson SP, Kjaer M (2021) Tendon blood flow, angiogenesis, and tendinopathy pathogenesis. Transl Sports Med 4(6):756–771. 10.1002/tsm2.280

[CR98] Mifune Y et al (2012) Therapeutic potential of anterior cruciate ligament-derived stem cells for anterior cruciate ligament reconstruction. Cell Transplant 21(8):1651–1665. 10.3727/096368912X647234 22732227

[CR99] Mifune Y et al (2013) Tendon graft revitalization using adult anterior cruciate ligament (ACL)-derived CD34+ cell sheets for ACL reconstruction. Biomaterials 34(22):5476–5487. 10.1016/j.biomaterials.2013.04.013 23632324

[CR100] Millett PJ, Hussain ZB, Fritz EM, Warth RJ, Katthagen JC, Pogorzelski J (2017) Rotator cuff tears at the musculotendinous junction: classification and surgical options for repair and reconstruction. Arthrosc Tech 6(4):e1075–e1085. 10.1016/j.eats.2017.03.023 PMC5621706 28970995

[CR101] Miyamoto RG, Bosco JA, Sherman OH (2009) Treatment of medial collateral ligament injuries. J Am Acad Orthop Surg 17(3):152–161. 10.5435/00124635-200903000-00004 19264708

[CR102] Morel M, Boutry N, Demondion X, Legroux-Gerot I, Cotten H, Cotten A (2005) Normal anatomy of the heel entheses: anatomical and ultrasonographic study of their blood supply. Surg Radiol Anat 27(3):176–183. 10.1007/s00276-004-0311-6 15917987

[CR103] Moschini, G. et al. 2026. *HIF1α gates tendon response to overload and drives tendinopathy independently of vascular recruitment*. https://www.science.org. 10.1126/scitranslmed.adt1228 41499520

[CR104] Nakano N et al (2015) Age-dependent healing potential of anterior cruciate ligament remnant-derived cells. Am J Sports Med 43(3):700–708. 10.1177/0363546514561436 25556219

[CR105] Niazi GE, Hassan MS, Elfawy DM (2020) Ultrasound-guided injection of platelet-rich plasma (PRP) in rotator cuff tendinopathy: effect on patients’ symptoms and supraspinatus tendon thickness. Egypt J Radiol Nucl Med 51(1):111. 10.1186/s43055-020-00221-2

[CR106] Nishimori M et al (2012) Role of angiogenesis after muscle derived stem cell transplantation in injured medial collateral ligament. J Orthop Res 30(4):627–633. 10.1002/jor.21551 21913220

[CR107] Noonan TJ, Garrett WE (1999) Muscle strain injury. J Am Acad Orthop Surg 7(4):262–269 10.5435/00124635-199907000-00006 10434080

[CR108] Ouyang HW, Goh JCH, Lee EH (2004) Use of bone marrow stromal cells for tendon graft-to-bone healing. Am J Sports Med 32(2):321–327. 10.1177/0095399703258682 14977654

[CR109] Petersen W, Tillmann B (1999) Structure and vascularization of the cruciate ligaments of the human knee joint. Anat Embryol 200(3):325–334. 10.1007/s004290050283 10463347

[CR110] Pneumaticos SG, Triantafyllopoulos GK, Chatziioannou S, Basdra EK, Papavassiliou AG (2011) Biomolecular strategies of bone augmentation in spinal surgery. Trends Mol Med 17(4):215–222 10.1016/j.molmed.2010.12.002 21195666

[CR111] Prodromos CC, Finkle S, Prodromos A, Chen JL, Schwartz A, Wathen L (2021) Treatment of rotator cuff tears with platelet rich plasma: a prospective study with 2 year follow‐up. BMC Musculoskelet Disord 22(1):499. 10.1186/s12891-021-04288-4 PMC8164813 34051761

[CR112] Pufe T, Petersen W, Tillmann B, Mentlein R (2001) The angiogenic peptide vascular endothelial growth factor is expressed in foetal and ruptured tendons. Virchows Arch 439(4):579–585. 10.1007/s004280100422 11710646

[CR113] Radice F, Yánez R, Gutiérrez V, Rosales J, Pinedo M, Coda S (2010) Comparison of magnetic resonance imaging findings in anterior cruciate ligament grafts with and without autologous platelet-derived growth factors. Arthroscopy 26(1):50–57. 10.1016/j.arthro.2009.06.030 20117627

[CR114] Rao R, Bhattacharyya R, Andrews B, Varma R, Chen A (2022) The management of combined ACL and MCL injuries: a systematic review. J Orthop 34:21–30. 10.1016/j.jor.2022.07.024 PMC9382135 35992613

[CR115] Rathbun JB, Macnab I (1970) The microvascular pattern of the rotator cuff. J Bone Joint Surg British Vol 52(3):540–553 5455089

[CR116] Recker AJ, Torres L, Dennis E, Scholten DJ, Waterman BR (2021) Latissimus dorsi tendon reconstruction at the myotendinous junction with acellular dermal allograft. Video J Sports Med. 10.1177/26350254211032671 PMC11887877 40308282

[CR117] Rha D, Park G-Y, Kim Y-K, Kim MT, Lee SC (2013) Comparison of the therapeutic effects of ultrasound-guided platelet-rich plasma injection and dry needling in rotator cuff disease: a randomized controlled trial. Clin Rehabil 27(2):113–122. 10.1177/0269215512448388 23035005

[CR118] Riggin CN et al (2021) Modulation of vascular response after injury in the rat Achilles tendon alters healing capacity. J Orthop Res 39(9):2000–2016. 10.1002/jor.24861 PMC7960560 32936495

[CR119] Riggin CN et al (2022) Increasing vascular response to injury improves tendon early healing outcome in aged rats. Ann Biomed Eng 50(5):587–600. 10.1007/s10439-022-02948-7 PMC9107615 35303172

[CR120] Roberts S, Colombier P, Sowman A, Mennan C, Rölfing JHD, Guicheux J, Edwards JR (2016) Ageing in the musculoskeletal system: cellular function and dysfunction throughout life. Acta Orthop 87:15–25. 10.1080/17453674.2016.1244750 PMC5389428 27748151

[CR121] Rudzki JR et al (2008) Contrast-enhanced ultrasound characterization of the vascularity of the rotator cuff tendon: age- and activity-related changes in the intact asymptomatic rotator cuff. J Shoulder Elbow Surg 17(1):S96–S100 10.1016/j.jse.2007.07.004 18069013

[CR122] Saether EE, Chamberlain CS, Leiferman EM, Kondratko-Mittnacht JR, Li WJ, Brickson SL, Vanderby R (2014) Enhanced medial collateral ligament healing using mesenchymal stem cells: dosage effects on cellular response and cytokine profile. Stem Cell Rev Rep 10(1):86–96. 10.1007/s12015-013-9479-7 PMC3946728 24174129

[CR123] Saether EE, Chamberlain CS, Aktas E, Leiferman EM, Brickson SL, Vanderby R (2016) Primed mesenchymal stem cells alter and improve rat medial collateral ligament healing. Stem Cell Rev Rep 12(1):42–53. 10.1007/s12015-015-9633-5 PMC4968884 26530282

[CR124] Sahin H, Tholema N, Petersen W, Raschke MJ, Stange R (2012) Impaired biomechanical properties correlate with neoangiogenesis as well as VEGF and MMP-3 expression during rat patellar tendon healing. J Orthop Res 30(12):1952–1957. 10.1002/jor.22147 22615070

[CR125] Salini V, Vanni D, Pantalone A, Abate M (2015) Platelet rich plasma therapy in non-insertional achilles tendinopathy: the efficacy is reduced in 60-years old people compared to young and middle-age individuals. Front Aging Neurosci. 10.3389/fnagi.2015.00228 PMC4674567 26696880

[CR126] Van Schaik KD, Lee KS (2021) Orthobiologics: diagnosis and treatment of common tendinopathies. Semin Musculoskelet Radiol 25(06):735–744. 10.1055/s-0041-1735475 34937114

[CR127] Schett G, Lories RJ, D’Agostino M-A, Elewaut D, Kirkham B, Soriano ER, McGonagle D (2017) Enthesitis: from pathophysiology to treatment. Nat Rev Rheumatol 13(12):731–741. 10.1038/nrrheum.2017.188 29158573

[CR128] Schmidt-Rohlfing B, Graf J, Schneider U, Niethard FU (1992) The blood supply of the Achilles tendon. Int Orthop. 10.1007/BF00182980 1572766

[CR129] Shi Z, Yao C, Shui Y, Li S, Yan H (2023) Research progress on the mechanism of angiogenesis in wound repair and regeneration. Front Physiol. 10.3389/fphys.2023.1284981 PMC10711283 38089479

[CR130] Soslowsky LJ, Thomopoulos S, Tun S, Flanagan CL, Keefer CC, Mastaw J, Carpenter JE (2000) Neer award 1999. Overuse activity injures the supraspinatus tendon in an animal model: a histologic and biomechanical study. J Shoulder Elbow Surg 9(2):79–84 10810684

[CR131] Stein V, Laprell H, Tinnemeyer S, Petersen W (2000) Quantitative assessment of intravascular volume of the human Achilles tendon. Acta Orthop Scand 71(1):60–63. 10.1080/00016470052943919 10743995

[CR132] Stein BE, Stroh DA, Schon LC (2015) Outcomes of acute Achilles tendon rupture repair with bone marrow aspirate concentrate augmentation. Int Orthop 39(5):901–905. 10.1007/s00264-015-2725-7 25795246

[CR133] van Sterkenburg MN, van Dijk CN (2011) Mid‐portion Achilles tendinopathy: why painful? An evidence‐based philosophy. Knee Surg Sports Traumatol Arthrosc 19(8):1367–1375. 10.1007/s00167-011-1535-8 PMC3136709 21567177

[CR134] Svensson RB, Heinemeier KM, Couppé C, Kjaer M, Magnusson SP (2016) Effect of aging and exercise on the tendon. J Appl Physiol 121(6):1353–1362. 10.1152/japplphysiol.00328.2016 27150831

[CR135] Takeuchi S, Rothrauff BB, Kanto R, Onishi K, Fu FH (2022) Superb microvascular imaging (SMI) detects increased vascularity of the torn anterior cruciate ligament. Knee Surg Sports Traumatol Arthrosc 30(1):93–101. 10.1007/s00167-021-06640-6 34121144

[CR136] Tang JB et al (2016) Basic FGF or VEGF gene therapy corrects insufficiency in the intrinsic healing capacity of tendons. Sci Rep 6(1):20643. 10.1038/srep20643 PMC4749961 26865366

[CR137] Tei K et al (2008) Administrations of peripheral blood CD34-positive cells contribute to medial collateral ligament healing via vasculogenesis. Stem Cells 26(3):819–830. 10.1634/stemcells.2007-0671 18192236

[CR138] Tempfer H, Wagner A, Gehwolf R, Lehner C, Tauber M, Resch H, Bauer HC (2009) Perivascular cells of the supraspinatus tendon express both tendon- and stem cell-related markers. Histochem Cell Biol 131(6):733–741. 10.1007/s00418-009-0581-5 19280209

[CR139] Tempfer H et al (2018) Bevacizumab improves Achilles tendon repair in a rat model. Cell Physiol Biochem 46(3):1148–1158. 10.1159/000489057 29672303

[CR140] Teng C, Zhou C, Xu D, Bi F (2016) Combination of platelet-rich plasma and bone marrow mesenchymal stem cells enhances tendon–bone healing in a rabbit model of anterior cruciate ligament reconstruction. J Orthop Surg Res 11(1):96. 10.1186/s13018-016-0433-7 PMC5015347 27605093

[CR141] Teunis T, Lubberts B, Reilly BT, Ring D (2014) A systematic review and pooled analysis of the prevalence of rotator cuff disease with increasing age. J Shoulder Elbow Surg 23(12):1913–1921. 10.1016/j.jse.2014.08.001 25441568

[CR142] Tidball JG (1983) The geometry of actin filament-membrane associations can modify adhesive strength of the myotendinous junction. Cell Motil 3(5):439–447 10.1002/cm.970030512 6607113

[CR143] Tohno Y et al (1999) Age-related changes of elements in human anterior cruciate ligaments and ligamenta capitum femorum. Biol Trace Elem Res 68(2):181–192. 10.1007/BF02784406 10327028

[CR144] Tol JL, Spiezia F, Maffulli N (2012) Neovascularization in Achilles tendinopathy: have we been chasing a red herring? Knee Surg Sports Traumatol Arthrosc 20(10):1891–1894. 10.1007/s00167-012-2172-6 22890896

[CR145] Tong S, Sun Y, Kuang B, Wang M, Chen Z, Zhang W, Chen J (2024) A comprehensive review of muscle–tendon junction: structure, function, injury and repair. Biomedicines 12(2):423 10.3390/biomedicines12020423 PMC10886980 38398025

[CR146] Tsechelidis OB, Sabido-Sauri R, Aydin SZ (2023) Enthesitis in spondyloarthritis including psoriatic arthritis—to inject or not to inject?: a narrative review. Clin Ther 45(9):852–859. 10.1016/j.clinthera.2023.08.002 37716837

[CR147] Turner NJ, Yates AJ, Weber DJ, Qureshi IR, Stolz DB, Gilbert TW, Badylak SF (2010) Xenogeneic extracellular matrix as an inductive scaffold for regeneration of a functioning musculotendinous junction. Tissue Eng Part A 16(11):3309–3317. 10.1089/ten.tea.2010.0169 20528669

[CR148] Uefuji A, Matsumoto T, Matsushita T, Ueha T, Zhang S, Kurosaka M, Kuroda R (2014) Age-related differences in anterior cruciate ligament remnant vascular-derived cells. Am J Sports Med 42(6):1478–1486. 10.1177/0363546514529092 24727934

[CR149] Usuelli FG, Grassi M, Maccario C, Vigano’ M, Lanfranchi L, Alfieri Montrasio U, de Girolamo L (2018) Intratendinous adipose-derived stromal vascular fraction (SVF) injection provides a safe, efficacious treatment for Achilles tendinopathy: results of a randomized controlled clinical trial at a 6-month follow-up. Knee Surg Sports Traumatol Arthrosc 26(7):2000–2010. 10.1007/s00167-017-4479-9 28251260

[CR150] Vaquette C, Slimani S, Kahn CJF, Tran N, Rahouadj R, Wang X (2010) A poly(lactic-co-glycolic acid) knitted scaffold for tendon tissue engineering: an in vitro and in vivo study. J Biomater Sci Polym Ed 21(13):1737–1760. 10.1163/092050609X12560455246676 20557686

[CR151] Vavken P, Saad FA, Murray MM (2010) Age dependence of expression of growth factor receptors in porcine ACL fibroblasts. J Orthop Res 28(8):1107–1112. 10.1002/jor.21111 PMC2892023 20186834

[CR152] Wallace CD, Amiel D (1991) Vascular assessment of the periarticular ligaments of the rabbit knee. J Orthop Res 9(6):787–791. 10.1002/jor.1100090603 1919840

[CR153] Wang IE, Mitroo S, Chen FH, Lu HH, Doty SB (2006) Age‐dependent changes in matrix composition and organization at the ligament‐to‐bone insertion. J Orthop Res 24(8):1745–1755. 10.1002/jor.20149 16779829

[CR154] Wang C, Zhang Y, Zhang G, Yu W, He Y (2021) Adipose stem cell-derived exosomes ameliorate chronic rotator cuff tendinopathy by regulating macrophage polarization: from a mouse model to a study in human tissue. Am J Sports Med 49(9):2321–2331. 10.1177/03635465211020010 34259608

[CR155] Watad A, Cuthbert RJ, Amital H, McGonagle D (2018) Enthesitis: much more than focal insertion point inflammation. Curr Rheumatol Rep 20(7):41. 10.1007/s11926-018-0751-3 PMC5976708 29846815

[CR156] Woo SL-Y, Vogrin TM, Abramowitch SD (2000) Healing and repair of ligament injuries in the knee. J Am Acad Orthop Surg 8(6):364–372. 10.5435/00124635-200011000-00004 11104400

[CR157] Xie X, Zhao S, Wu H, Xie G, Huangfu X, He Y, Zhao J (2013) Platelet-rich plasma enhances autograft revascularization and reinnervation in a dog model of anterior cruciate ligament reconstruction. J Surg Res 183(1):214–222. 10.1016/j.jss.2013.01.020 23472861

[CR158] Xu H et al (2023) Bioactive glass-elicited stem cell-derived extracellular vesicles regulate M2 macrophage polarization and angiogenesis to improve tendon regeneration and functional recovery. Biomaterials. 10.1016/j.biomaterials.2023.121998 36641814

[CR159] Yan Z, Yin H, Nerlich M, Pfeifer CG, Docheva D (2018) Boosting tendon repair: interplay of cells, growth factors and scaffold-free and gel-based carriers. J Exp Orthop 5(1):1. 10.1186/s40634-017-0117-1 PMC5768579 29330711

[CR160] Yoshikawa T et al (2006) Effects of local administration of vascular endothelial growth factor on mechanical characteristics of the semitendinosus tendon graft after anterior cruciate ligament reconstruction in sheep. Am J Sports Med 34(12):1918–1925. 10.1177/0363546506294469 17092923

[CR161] Yu JS, Popp JE, Kaeding CC, Lucas J (1995) Correlation of MR imaging and pathologic findings in athletes undergoing surgery for chronic patellar tendinitis. AJR Am J Roentgenol 165(1):115–118. 10.2214/ajr.165.1.7785569 7785569

[CR162] Zamboulis DE, Marr N, Lenzi L, Birch HL, Screen HRC, Clegg PD, Thorpe CT (2024) The interfascicular matrix of energy storing tendons houses heterogenous cell populations disproportionately affected by aging. Aging Dis 15(1):295 10.14336/AD.2023.0425-1 PMC10796100 37307816

[CR163] Zhang F et al (2003) Effect of vascular endothelial growth factor on rat Achilles tendon healing. Plast Reconstr Surg 112(6):1613–1619. 10.1097/01.PRS.0000086772.72535.A4 14578792

[CR164] Zhang J, Yuan T, Wang JH-C (2016) Moderate treadmill running exercise prior to tendon injury enhances wound healing in aging rats. Oncotarget 7(8):8498–8512. 10.18632/oncotarget.7381 PMC4890982 26885754

[CR165] Zhang M, Zhen J, Zhang X, Yang Z, Zhang L, Hao D, Ren B (2019) Effect of autologous platelet-rich plasma and gelatin sponge for tendon-to-bone healing after rabbit anterior cruciate ligament reconstruction. Arthroscopy 35(5):1486–1497. 10.1016/j.arthro.2018.11.014 30979627

[CR166] Zhu T, Zhou J, Hwang J, Xu X (2022) Effects of platelet-rich plasma on clinical outcomes after anterior cruciate ligament reconstruction: a systematic review and meta-analysis. Orthop J Sports Med. 10.1177/23259671211061535

